# The ice phenology as a predictor of *Planktothrix rubescens* bloom in vegetation season in temperate lakes

**DOI:** 10.3389/fmicb.2024.1384435

**Published:** 2024-06-26

**Authors:** Tomasz Lenard, Wojciech Ejankowski

**Affiliations:** ^1^Department of Animal Physiology and Toxicology, Faculty of Medicine, The John Paul II Catholic University of Lublin, Lublin, Poland; ^2^Laboratory of Research and Nature Protection, Krzczonów, Poland

**Keywords:** *Planktothrix rubescens*, cyanobacterial blooms, vertical distribution, physical and chemical parameters of water, ice phenology, climatic conditions

## Abstract

**Introduction:**

Global warming affects air and water temperatures, which impacts the phenology of lakes and aquatic ecosystems. These changes are most noticeable during winter, when the potentially toxic *Planktothrix rubescens* forms its inoculum for annual blooms. Mostly, research has been conducted on alpine lakes, where blooms have persisted for decades, while a few have focused on temperate lakes. Our study aimed to determine the factors influencing the dynamics of the development of *P. rubescens* in temperate lakes where blooms occasionally occur, with a particular emphasis on the role of ice phenology.

**Methods:**

We investigated the vertical distribution of *P. rubescens* in an annual cycle in three temperate lakes. Samples were collected monthly in the winter and biweekly during the vegetative seasons. Overall, 434 samples were collected and analyzed according to biological and chemical parameters. Physical parameters were measured *in situ*.

**Results:**

The vegetation seasons in temperate lakes showed a similar development pattern in the *P. rubescens* population as that in alpine lakes. Our results also show the influence of physical and chemical factors on the vertical distribution of this cyanobacterium. These results revealed the significant impact of *P. rubescens* filaments on phytoplankton biodiversity and biomass. Our data show the role of ice phenology in the establishment of the winter inoculum of *P. rubescens* and its further mass development until its disappearance in autumn.

**Conclusion:**

A climate-zone-independent pattern of *P. rubescens* blooms was observed during the vegetation periods. The population of *P. rubescens* was more influenced by physical factors than by the availability of dissolved nutrients in the water. Despite the same etiology, global warming has been shown to cause different responses in aquatic ecosystems, which affect the different nature of *P. rubescens* appearances. We associated blooms in temperate lakes, in contrast to alpine lakes, mainly with the presence of ice cover during severe winters, when the species establishes its inoculum. Hence, blooms in temperate lakes occur at different time intervals. Therefore, the dynamics of periodic blooms of *P. rubescens* in temperate lakes provide novel knowledge to the case study and a counterpoint to permanent blooms found in deep alpine lakes.

## 1 Introduction

Lakes are generally considered to be great sentinels for global change. Their relatively small scale enables observing rapid responses to environmental changes, which may translate to larger aquatic ecosystems (Hessen et al., 2009). A worldwide warming trend is reflected in increasing air and water temperatures, which directly affect seasonal anomalies, changes in the thermal regime of lakes, and, in turn, all biological processes in the aquatic environment (Dokulil, 2016; Lenssen et al., 2019; Kraemer et al., 2021; Woolway et al., 2020; Weiskopf et al., 2020; GISTEMP Team, 2024). This trend strongly supports the development of cyanobacteria in warm surface waters, where they often cause mass appearances called blooms (Paerl and Huismann, 2008; Ho et al., 2019). Most of these blooms occur because of the ability of cyanobacteria to grow under high light intensity and nutrient availability (Huisman et al., 2018). Therefore, phylogenetically old cyanobacteria have developed many environmental and physiological adaptations, such as the formation of filamentous or coccal colonies, heterocysts, production of mucilage and bioactive metabolites, which help them survive and outcompete other phytoplankton species in surface waters (Carey et al., 2012; Nandagopal et al., 2021).

Nevertheless, in this highly diverse phytoplankton group, species are also able to develop in lower, metalimnetic, or hypoloimnetic layers of water using other adaptations, such as the formation of gas vesicles (aerotopes), or using other pigments rather than green chlorophyll, to perform photosynthesis under low light intensities (Micheletti et al., 1998; Walsby and Schanz, 2002; Chorus and Welker, 2021). Hence, cyanobacteria can also survive in lower water columns or even cause blooms responsible for a deep chlorophyll maximum (DCM) phenomenon in deep lakes (Leach et al., 2018). From this perspective, the presence of cyanobacterial blooms in the metalimnion may sometimes be difficult to discover or even omitted, for example, in lake monitoring in some countries of the European Union, if the lower water column is not considered (Pasztaleniec, 2016; Lenard and Poniewozik, 2022).

*Planktothrix rubescens* (Gomont) Anagnostidis & Komárek is one of the most studied filamentous cyanobacteria that develop in deep-water layers, often causing DCM (Walsby and Schanz, 2002; Jacquet et al., 2005; Legnani et al., 2005; Yankova et al., 2016; Fernández Castro et al., 2021; Knapp et al., 2021). This species has a group of aerotopes that improve its buoyancy and support its vertical migration in lentic waters (Komárek and Anagnostidis, 2008; D’Alelio et al., 2011). This ability helps avoid high light intensity, light flashes, and vertical mixing of water, to which *P. rubescens* is vulnerable (Davis et al., 2003a; Oberhaus et al., 2007). As a consequence, it produces an additional photosynthetic pigment, phycoerythrin, which belongs to the phycobilins (Schanz, 1986; Davis et al., 2003b; Komárek and Komárková, 2004), which enables photosynthesis in dim light in the presence of blue or green light with high photosynthetic efficiency (Micheletti et al., 1998; Kromkamp et al., 2001; Walsby et al., 2001; Oberhaus et al., 2007). *P. rubescens* is a cyanobacterium devoid of heterocysts and must utilize biogenic compounds dissolved in water (Komárek and Komárková, 2004), which might limit its development. Nevertheless, its allelopathic capacity, manifested by its ability to produce toxins and other secondary metabolites, hampers the development of other primary producers, and it uses the entire pool of available nutrients in the water (Feuillade, 1994; Oberhaus et al., 2008; Salmaso et al., 2013; Kurmayer et al., 2016). Therefore, *P. rubescens* can develop well (Chorus et al., 2011) even with relatively low concentrations of dissolved nutrients in water, because of its ability to utilize organic forms of nitrogen and phosphorus or store nutrients in its cells (Feuillade et al., 1990; Reynolds, 2006; Kurmayer et al., 2016). Hence, its dense blooms in nutrient-poor metalimnion of deep and clear waters are mainly recorded in the mountain, alpine, and pre-alpine lakes (Micheletti et al., 1998; Walsby and Schanz, 2002; Jacquet et al., 2005; Legnani et al., 2005; Ernst et al., 2009; D’Alelio et al., 2011; Dokulil and Teubner, 2012; Hingsamer et al., 2014; Yankova et al., 2016; Copetti et al., 2017; Fernández Castro et al., 2021; Knapp et al., 2021; Carratalà et al., 2023), and less frequently in volcanic lakes (Messineo et al., 2006; Messyasz et al., 2012) and lowland temperate lakes (Wojciechowska and Krupa, 1992; Krupa and Czernaś, 2003; Padisák et al., 2003; Salmaso and Padisák, 2007; Lenard, 2009; Lenard, 2015; Lenard and Ejankowski, 2017; Lenard and Poniewozik, 2022; Kröger et al., 2023) or occasionally in lakes in semiarid Mediterranean climate (Naselli-Flores et al., 2007). Regardless of the location of the lakes, their common characteristics are great depth, the presence of stable stratification during summer providing an adequate light climate and a low-temperature gradient in the metalimnion, and the availability of nutrients. The recent studies from alpine lakes suggest also the positive effect of global warming on the existence and development of overwintering population of *P. rubescens* that constitute the first step for its annual development (Yankova et al., 2017; Knapp et al., 2021; Moiron et al., 2021; Carratalà et al., 2023). In contrast, some reports from temperate lakes suggest that *P. rubescens* blooms may have been influenced by “unusual” event of long lasting periods of ice in the year before the bloom occurred (Padisák et al., 2003).

Based on the permanent blooms of *P. rubescens* in alpine lakes, its mass development in temperate lakes is occasional and often restricted to short periods. Therefore, in this study, we aimed to (1) determine the dynamics of the annual bloom of *P. rubescens* that occurred in three lakes in the temperate zone of eastern Poland, and (2) determine the reason for the appearance of the bloom at different time intervals. We hypothesize that the ice phenology coupled with the presence of severe winter is the main reason for the establishment of the winter inoculum of *P. rubescens* in temperate lakes, which is crucial for its further mass development during the growing season.

## 2 Materials and methods

The study was carried out in three lakes, Rogóźno (51°22′ N 22°58′ E, 167.7 m a.s.l), Krasne (51°25′ N 22°57′ E, 164.0 m a.s.l), and Piaseczno (51°23′ N, 23°01′ E, 170.6 m a.s.l), located in the Łęczna-Włodawa Plain in eastern Poland, which is a part of the large cross-border Polesie region (Kondracki, 2002). All lakes were deep, dimictic, and mesotrophic. The maximum depth and lake area were 25.4 m and 57.1 ha in Lake Rogoźno, 33.0 m and 75.9 ha in Lake Krasne, and 38.8 m and 84.7 ha in Lake Piaseczno (Harasimiuk et al., 1998). The water level in Lake Piaseczno had highly fluctuated, at 1.68 m in the last 20 years. Moreover, it was estimated to decrease at a rate of *ca.* 1 cm per year, which is connected to a decrease in water resources in the chalk layer of the Lublin Upland and directions of underground outflow changes from the catchment area of the upper Wieprz River (Michalczyk et al., 2017).

The phytoplankton biomass, chlorophyll-*a* concentration, as well as physical and chemical parameters of water, were studied during the annual appearance of *P. rubescens*, which lasted from January to September of 2006, 2010, and 2014 in lakes Rogóźno, Krasne and Piaseczno, respectively. Water samples for analyses were collected monthly in winter (January–March) and biweekly during the vegetation periods (April–September) from the deepest part of the lakes using a Ruttner-type water sampler (2.0 L capacity). A 3 liters samples of water were always taken from a depth of 0.5 m, from 1 m to 8 m in Lakes Rogóźno and Krasne, or from 1 m to 12 m in Lake Piaseczno, at one-meter intervals. Thereby, we always received nine (in lakes Rogóźno and Krasne) or 13 (in Lake Piaseczno) samples in every sampling date, giving a total of 126, 126, and 182 samples for lakes Rogóźno, Krasne, and Piaseczno, respectively.

In the laboratory, the water samples of 1 to 2 liters were filtered through Whatman GF/C microfiber filter with a particle retention of 1.2 μm. Next, the filters were frozen for further analyses of chlorophyll-*a*, and the filtered water was immediately used for analyses of dissolved nitrogen and phosphorus compounds. Unfiltered samples of water, after mineralization of organic matter, were used for determination of total nitrogen and phosphorus content. The samples were analyzed using spectrophotometric methods to determine the concentrations of chlorophyll-*a* with use of ethanol(Nusch, 1980), inorganic (P–PO_4_) and total phosphorus (TP) with use of ammonium molybdate, and inorganic (N–NO_3_ and N–NH_4_) and total nitrogen (TN), with use of chromotropic acid or Nessler’s reagent (Hermanowicz et al., 1999). A 100 mL samples of water for phytoplankton analyses were fixed with Lugol’s iodine solution and a formalin–glycerin mixture. The abundance of phytoplankton was determined according to the standard Utermöhl method (Utermöhl, 1958), and the algal biovolume was calculated using the formula described by Hillebrand et al. (1999). The fixed water samples were transferred to a 5–50 mL settling chamber. After sedimentation, the algal abundance was evaluated using an inverted microscope (Zeiss Axiovert 135). Small phytoplankton species on the belts were counted in each chamber, whereas larger forms (filamentous or coccal colonies) were counted at the entire bottom of the chamber. The unit length of 100 μm and a surface of 300 μm^2^ were one individual for filamentous and coccal colonies, respectively. Additionally, the samples for taxonomic analyses of phytoplankton were collected using a plankton net (20 μm mesh size) and were left without fixation to observe live specimens under a light microscope (Nikon Eclipse 80i). If possible, all phytoplankton organisms in the samples were identified to the species level. To determine the differences in phytoplankton species composition, the Shannon-Wiener diversity (Shannon and Wiener, 1963) and Pielou’s evenness indices (Pielou, 1975) were calculated based on the abundance of the phytoplankton community.

Physical parameters such as water temperature (to calculate the range of the mixing zone, Z_mix_), water transparency by Secchi disc visibility (SD), and photosynthetic active radiation (PAR), with the use of Li-Cor LI-250A light meter equipped with LI-192SA underwater quantum flat sensor were measured *in situ*. Additionally, the values of PAR were used to calculate the euphotic zone (Z_eu_) range precisely. The mean air temperature in the study area was calculated using data from a meteorological station in Włodawa (Polesie region) obtained from an online service.^1^

Correlations between abiotic (physical and chemical) and biotic (phytoplankton biomass and chlorophyll-*a* concentration) parameters and *P. rubescens* biomass were evaluated using Spearman’s rank correlation test (Sokal and Rohlf, 1995). The same test was used to analyze the relationship between the appearance of *P. rubescens* and air temperature in January–March. All calculations were performed using the PSPP (ver. 2.0.0) package.

## 3 Results

### 3.1 The effect of environmental conditions on *Planktothrix rubescens* development in winter

**Figure 1:**
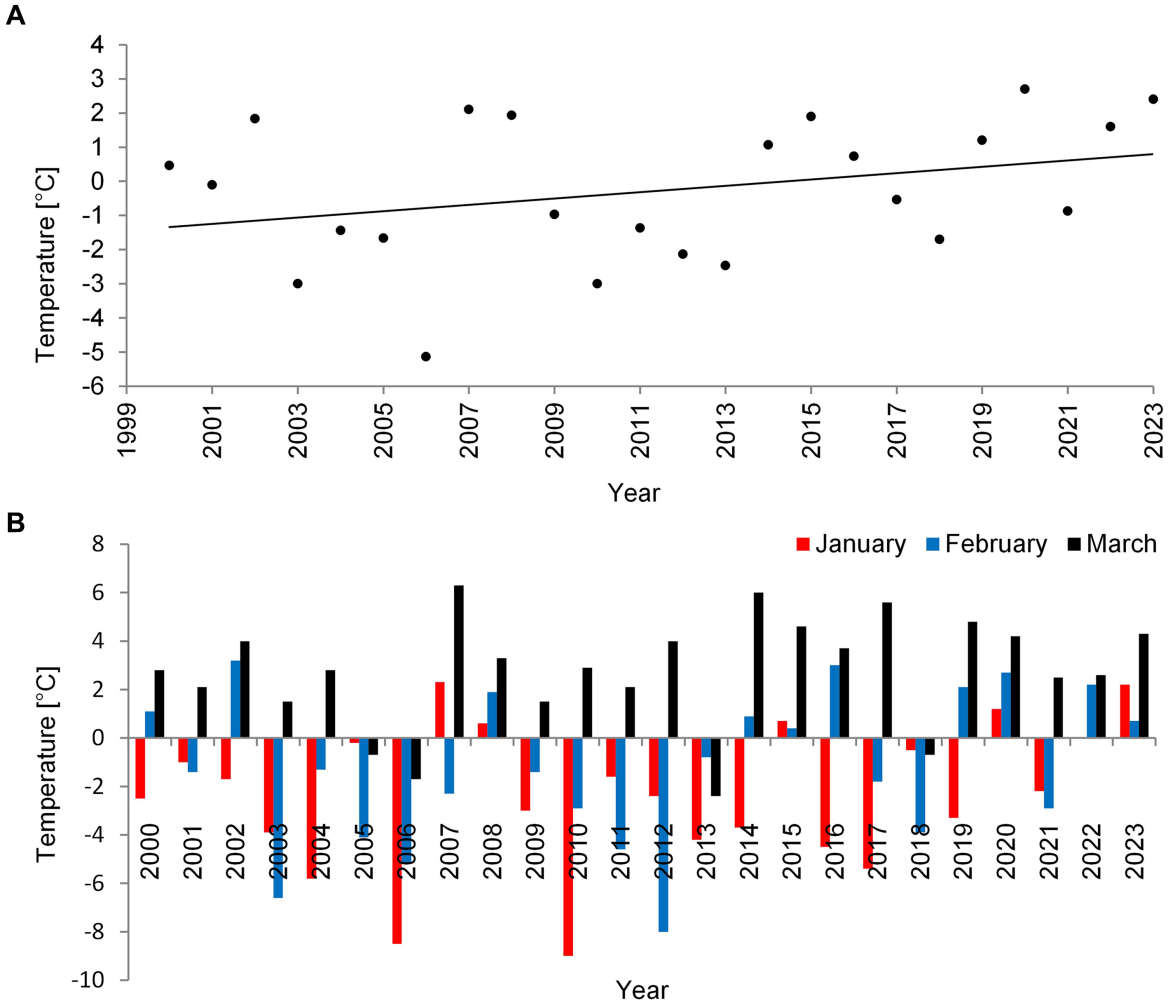
Mean air temperature values in °C for the winter (January–March) **(A)** and mean temperature values in °C in a particular month: January, February, and March **(B)** in the Łęczna-Włodawa Plain for 24 years.

During the last 24 years (2000–2023), in which we monitored three deep lakes of Łęczna-Włodawa Plain, we noticed a certain increase in mean air temperature in winter (Figure 1A). During this time, *P. rubescens* found favorable conditions and started to develop only during the three winter periods of 2006, 2010, and 2014. The climatic conditions during winters with *P. rubescens* were generally similar. However, the bloom always appeared only in one lake each year, in Lake Rogóźno, Krasne or Piaseczno in 2006, 2010, and 2014, respectively. The minimum, maximum, and mean temperatures of air in January in these years were, on average, several degrees lower than those in the years without its appearance, for example, the mean air temperature in January was −7°C, while it was only −1.8°C in the periods without its development (Supplementary Figure S1). A high correlation indicated the strong link between air temperatures in January 2000–2023 and the occurrence of *P. rubescens* in the studied lakes (Spearman’s test, *r*_s_ = 0.5, *p* < 0.05). In contrast, this effect was statistically insignificant, *r*_s_ = −0.18 and *r*_s_ = 0.01, *p* < 0.05 in February and March, respectively. Two of the studied winters (2006 and 2010) were extremely cold, with the mean air temperature for January close to −9°C (Figure 1B) and the lowest recorded temperature close to −30°C. Conversely, the mean air temperature in January in the last winter with *P. rubescens* appearance (2014) was *ca.* –4°C (Figure 1B), however, still with the lowest recorded temperature close to −21°C.

**Figure 2:**
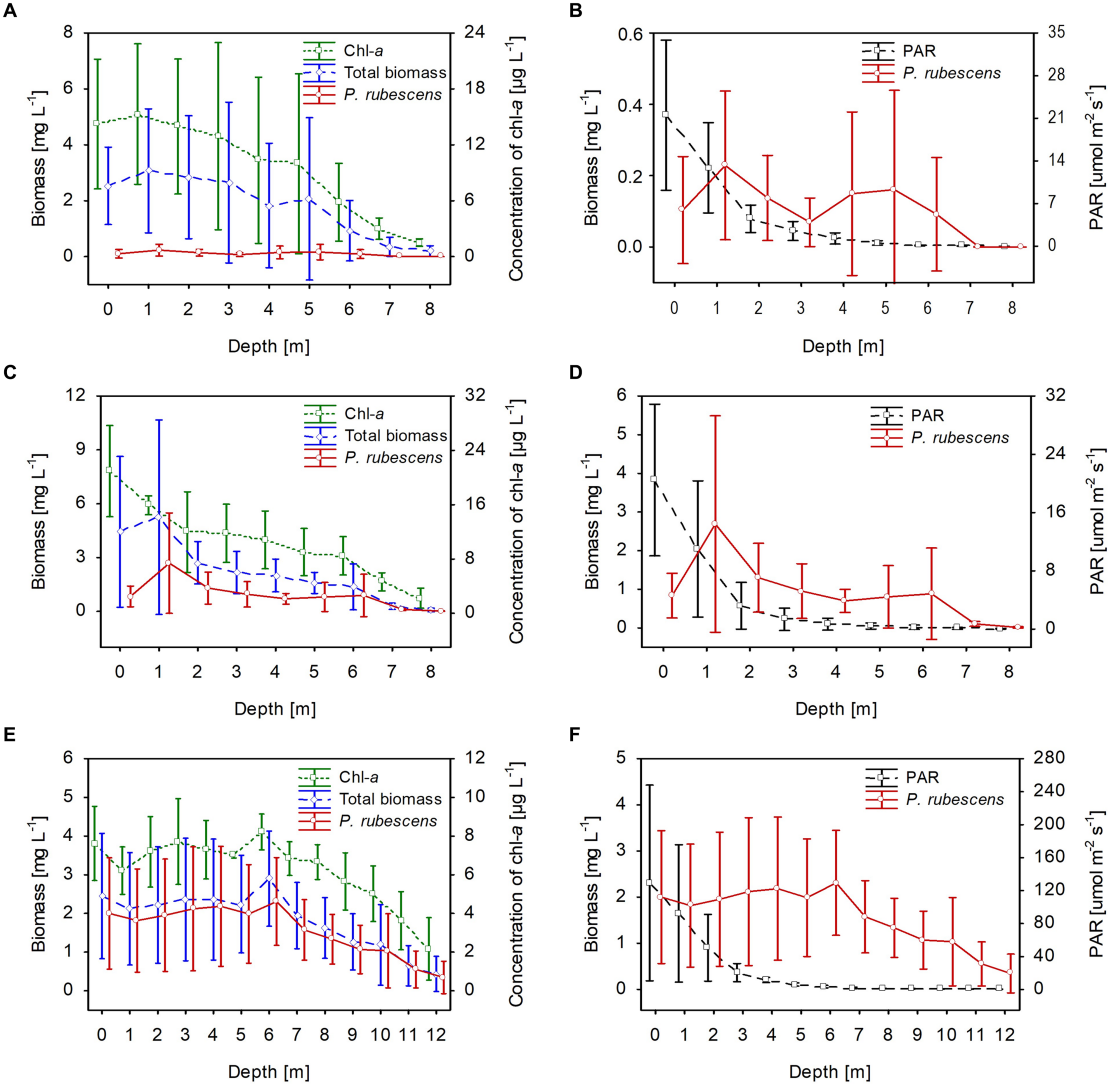
Vertical distribution of mean values (±standard deviations) of selected biological and physical parameters of water during winter in Lake Rogóźno in 2006 **(A,B)**; in Lake Krasne in 2010 **(C,D)**; in Lake Piaseczno in 2014 **(E,F)**. Chl-a, concentration of chlorophyll-a; PAR, photosynthetic active radiation; total biomass, the total phytoplankton biomass.

**Figure 3:**
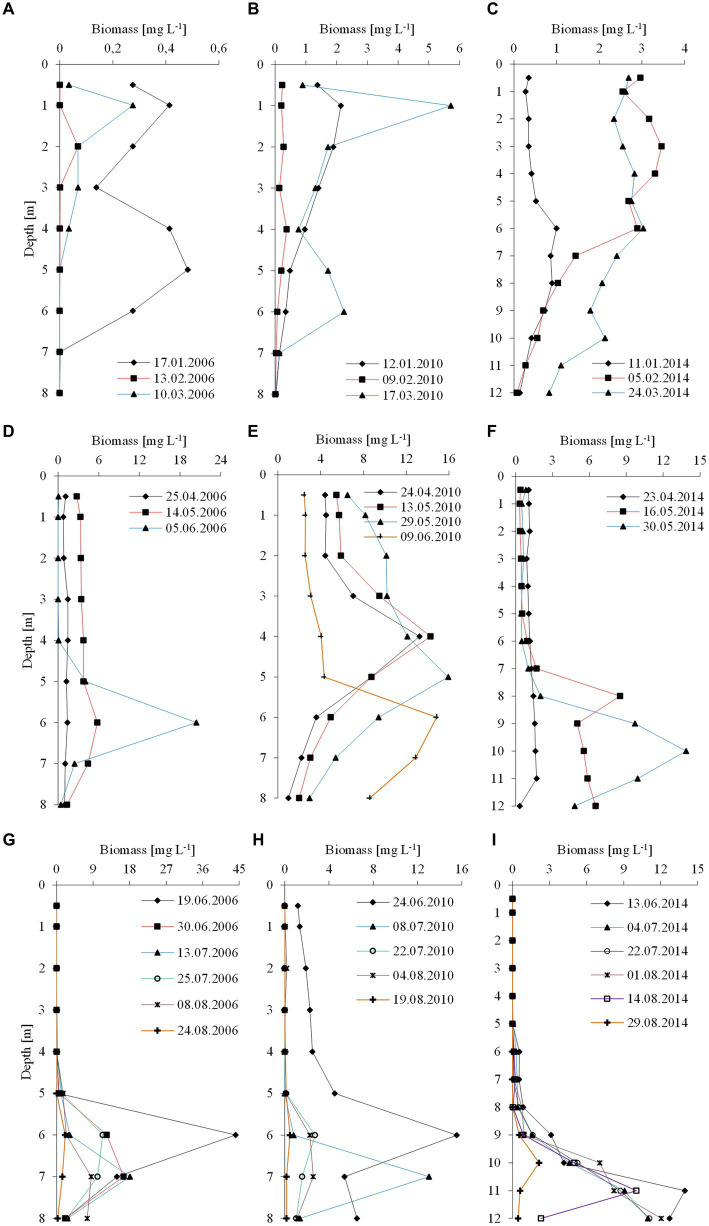
Vertical distribution of the biomass of *P. rubescens* during winter season (upper panel), spring season (middle panel) and summer season (lower panel) in the three studied lakes: **(A,D,G)** Rogóźno in 2006; **(B,E,H)** Krasne in 2010; and **(C,F,I)** Piaseczno in 2014.

The climatic conditions recorded during these three winters affected lake functioning (Table 1). In severe winters (2006 and 2010), the duration of ice cover was almost 4 months, from mid-December to mid-April. The consequence of such long-lasting ice cover was great variability in snow thickness (0–0.18 m) and ice cover (0.11–0.45 m). These conditions caused water column stability and significantly reduced the amount of light (PAR) penetrating the water available to phytoplankton (Table 1). Hence, the snow/ice cover protected the water below from freezing and supported the existence of strong inverse thermal stratification, i.e., in January 2006, during sampling day in Lake Rogóźno with air temperature −22.1°C, the temperature of water below 0.25 m of ice layer was *ca.* 1.4°C and was similar to these noted in March 2010 in Lake Krasne under 0.1 m of snow and 0.35 m of ice (Table 1). The light passing through the thick snow and ice cover (thickness, *ca.* 0.5 m) often absorbed more than 90% of incoming light (February and March 2006 and 2010, Table 1). In these conditions, the highest mean values of the phytoplankton biomass (2.5–5.5 mg L^−1^) and chlorophyll-*a* (14–22 μg L^−1^) were noted mainly at a depth of 0.5 to 2–3 m (Figures 2A–D). In both lakes (Rogóźno and Krasne), high shares in the total phytoplankton biomass had mixotrophic species belonging to cryptomonads – *Cryptomonas marssonii* Skuja and *Cryptomonas curvata* Ehrenberg; however, the shares of *P. rubescens* in Lake Rogóźno in 2006 were low, while its shares in Lake Krasne in 2010 often exceeded 60% in the total phytoplankton biomass. Nevertheless, high light absorption fostered the development of *P. rubescens* under thick snow/ice cover by creating stable, dim light conditions in the water (Figures 2B,D). Simultanously, the vertical distribution of *P. rubescens* biomass was the highest in both lakes (Rogóźno and Krasne) at a depth of 1 m, and decreased sharply with increasing depth (Figures 3A,B). However, in some cases, such as in February 2014 in Lake Piaseczno, the lack of snow cover and the presence of a thick layer (0.21 m) of so-called “black ice” devoid of air bubbles and almost perfectly transparent allowed more than 50% of the light reaching the surface to penetrate below the ice cover (Table 1). Under this highly transparent ice, the vertical distribution of *P. rubescens* biomass was almost homogenous at a depth of 0.5–6 m (*ca.* 3 mg L^−1^, Figure 3C). This trend in its biomass distribution in the gradient of light was also maintained during mixing periods in winter (samples taken at 11^th^ January and 24^th^ March 2014), which resulted in higher mean values of chlorophyll-*a* (*ca.* 7 μg L^−1^) and phytoplankton biomass (*ca.* 2.5 mg L^−1^) at a depth of 0.5–6 m than in the deeper water column, 7–12 m (Figures 2E,F). Even if more than 60% of the light penetrated the water during mixing time, its amount just under the water surface was still minor (the values of PAR slightly above 100 μmol m^−2^ s^−1^, Table 1) that still supported homogenous distribution of *P. rubescens* biomass to the depth of 6 m (Figure 3C).

**Table 1:** Physical parameters measured in winter periods in Lake Rogóźno, Krasne, and Piaseczno in 2006, 2010, and 2014, respectively.

Year	Month	Temperature [°C] in a sampling day		Thickness of		PAR [μmol m^−2^ s^−1^]		Light absorption by the snow and ice [%]	Approximate duration of ice cover	
		of air	of water beneath the ice	snow[m]	ice [m]	above the ice	beneath the ice		beginning	breakup
2006	J	−22.1	1.4	0	0.25	204.29	43.51	78.7	23rd Dec. 2005	15th Apr. 2006
	F	−12.8	1.0	0.18	0.33	491.63	14.25	97.2		
	M	−16.2	0.9	0.05	0.45	386.21	8.95	97.7		
2010	J	−15.0	1.4	0.07	0.11	222.15	36.32	83.7	15th Dec. 2009	1st Apr. 2010
	F	−12.3	1.8	0.14	0.24	514.89	28.64	94.5		
	M	−6.0	1.6	0.10	0.35	841.14	75.55	91.1		
2014	J	1.8	3.3^a^	-	-	167.82	124.11^a^	26.0 ^a^	19th Jan. 2014	14th Mar. 2014
	F	−10.6	1.3	0	0.21^b^	841.62	446.91	46.9		
	M	6.9	5.5^a^	-	-	170.81	108.71^a^	36.7 ^a^		

PAR, photosynthetic active radiation; J, F, M, January, February, March. ^a^The temperature or light just below the water surface; ^b^The presence of “black ice.”

**Figure 4:**
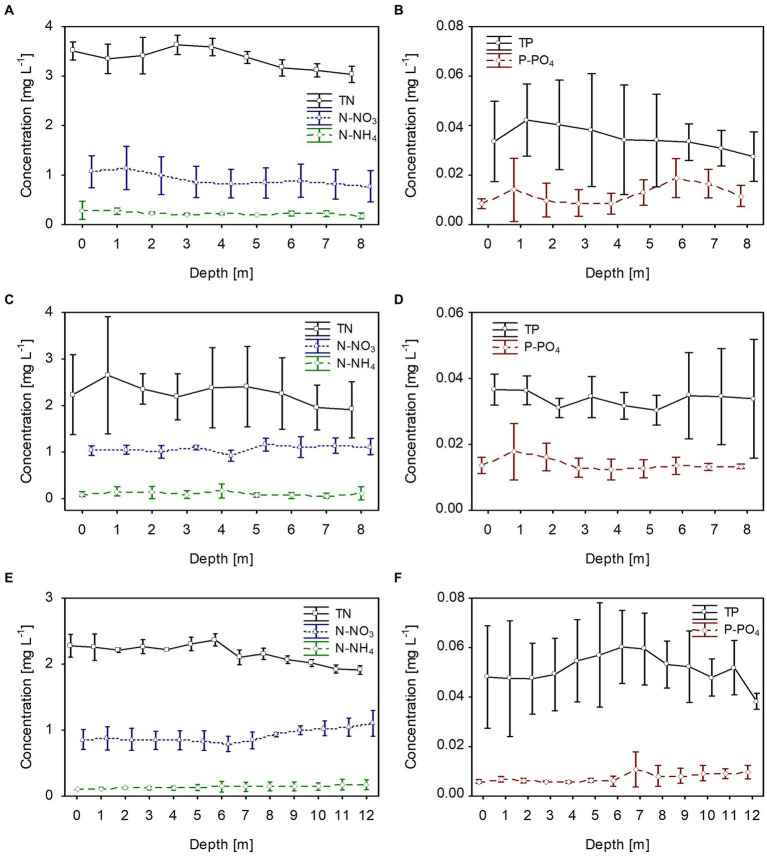
Vertical distribution of mean values (±standard deviations) of selected chemical parameters of water during winter in Lake Rogóźno in 2006 **(A,B)**; in Lake Krasne in 2010 **(C,D)**; in Lake Piaseczno in 2014 **(E,F)**. N-NH_4_, concentration of ammonium nitrogen; N-NO_3_, concentration of nitrate nitrogen; TN, concentration of total nitrogen; P-PO_4_, concentration of phosphate phosphorus; TP, concentration of total phosphorus.

The dissolved and total fractions of biogenic compounds (nitrogen and phosphorus) corresponded to the phytoplankton biomass distribution in all studied lakes. The mean values of dissolved nitrogen (N-NH_4_ and N-NO_3_) and phosphorus (P-PO_4_) were generally homogeneously distributed in the water column; however, there was a slight increase at greater depths, at which the phytoplankton biomass was lower (Figure 4). The mean values of dissolved fractions varied from 0.05 to 0.3 mg L^−1^, from 0.8 to 1.0 and from 0.008 to 0.017 for N-NH_4_, N-NO_3_ and P-PO_4_, respectively. Contrarily, total nitrogen (TN) values were slightly higher in the upper water layers with high phytoplankton biomass. They varied from 3.2 to 3.55 mg L^−1^, from 2.0 to 2.6 mg L^−1^, and from 2.0 to 2.4 mg L^−1^, in lakes Rogóźno, Krasne, and Piaseczno, respectively (Figures 4A,C,E). A similar trend was noted with the values of total phosphorus (TP), with mean values in the range of 0.03–0.04 mg L^−1^ in lakes Rogóźno and Krasne and 0.04–0.06 mg L^−1^ in Lake Piaseczno (Figures 4B,D,F).

### 3.2 *Planktothrix rubescens* development in the vegetative season before the onset of thermal stratification

The appearance of *P. rubescens* in winter predicted its mass development in the months following a given year. The spring season, lasting from the end of March (30^th^ March) to early June (9^th^ June), was the time of permanent water mixing in the studied lakes and the first step in the onset of thermal stratification. During permanent water mixing (23rd–25th April of the individual years), the biomass of *P. rubescens* was generally evenly distributed throughout the water column in lakes Rogóźno and Piaseczno, except of Lake Krasne, in which the peak of biomass was stated on the depth of 4 m (Figures 3D–F). However, over time, the temperature of the upper water layers increased, leading to the slow establishment of thermal stratification, and its biomass increased significantly in the deeper water layers of all lakes. These increases were particularly noticeable at the end of spring periods (29th May–9th June) when the highest values of *P. rubescens* biomass (often above 10 mg L^−1^) were found at the depths of 6 m, 5–6 m, and 8–12 m in Lake Rogóźno in 2006, Lake Krasne in 2010 and Lake Piaseczno in 2014, respectively (Figures 3D–F).

### 3.3 *Planktothrix rubescens* development during summer stratification

**Figure 5:**
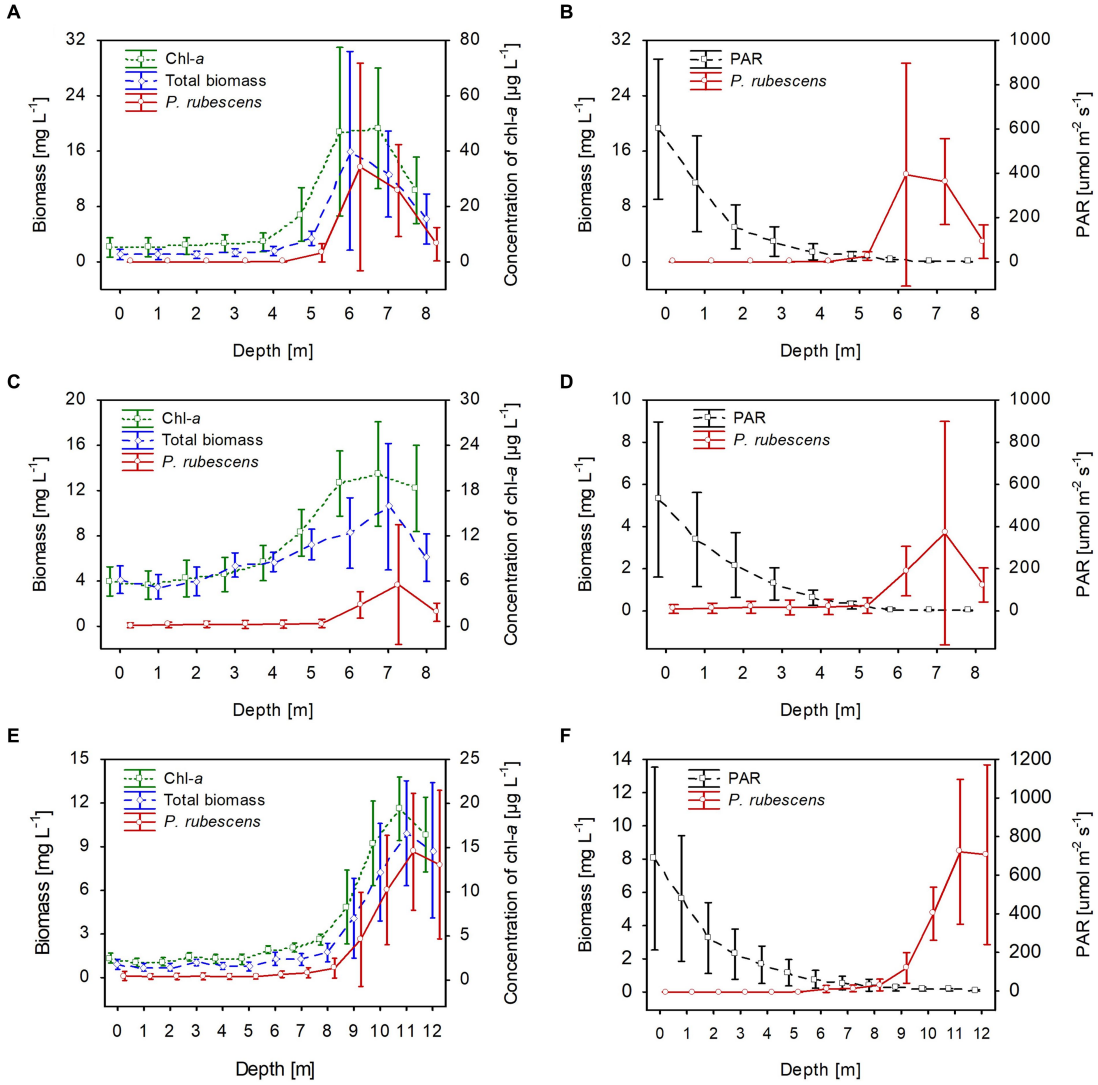
Vertical distribution of mean values (±standard deviations) of selected biological and physical parameters of water during summer thermal stratification in Lake Rogóźno in 2006 **(A,B)**; in Lake Krasne in 2010 **(C,D)**; in Lake Piaseczno in 2014 **(E,F)**. Chl-a, concentration of chlorophyll-a; PAR, photosynthetic active radiation; total biomass, the total phytoplankton biomass.

Stable thermal stratification persisted in the studied lakes from 13^th^ June to August. The metalimnion layer reached 4–8 m in lakes Rogóźno and Krasne and 7–12 m in Lake Piaseczno. The total biomass of phytoplankton in the metalimnion, in which the light intensities were extremely low (mean values of PAR mainly below 40 μmol m^−2^ s^−1^ that constituted less than 3% of surface light intensities), was almost completely dominated by *P. rubescens*. In contrast, its biomass in the epilimnion was negligible (Figure 5). The mean values of *P. rubescens* biomass and the concentration of chlorophyll-*a* were generally the highest in the middle of the metalimnion layer, giving deep chlorophyll maximum, i.e., in Lake Rogóźno at a depth of 6 m (13.8 mg L^−1^ and 44.8 μg L^−1^), in Lake Krasne at a depth of 7 m (3.9 mg L^−1^ and 20.4 μg L^−1^), and in Lake Piaseczno at a depth of 11 m (8.5 mg L^−1^ and 19.5 μg L^−1^, Figures 5A,C,E). The share of *P. rubescens* often exceeded 80% in the total biomass of phytoplankton in lakes Rogóźno and Piaseczno, while in Lake Krasne, this species co-occurred with the biomass of other filamentous cyanobacteria, i.e., *Limnothrix planctonica* (Woloszynska) Meffert and *Planktolyngbya limnetica* (Lemmermann) Komarkova-Legnerova & Cronberg that explains its lower, still above 50% shares in the total biomass of phytoplankton in the metalimnion (Figure 5C). Additionally, Lake Krasne was the only lake in the epilimnion of which higher values of total phytoplankton biomass (mean values *ca.* 4 mg L^−1^) were found. These water layers were dominated by the filamentous cyanobacteria *Aphanizomenon gracile* Lemmermann and the green alga *Closterium acutum* var. *variabile* (Lemmermann) W. Krieger.

**Figure 6:**
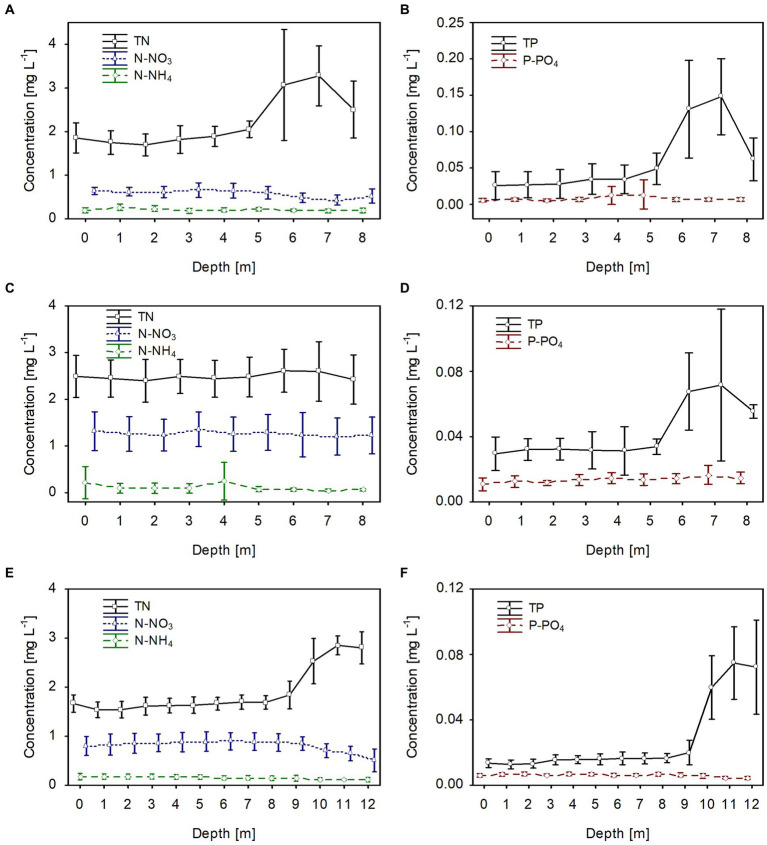
Vertical distribution of mean values (±standard deviations) of selected chemical parameters of water during summer thermal stratification in Lake Rogóźno in 2006 **(A,B)**; in Lake Krasne in 2010 **(C,D)** in Lake Piaseczno in 2014 **(E,F)**. N-NH_4_, concentration of ammonium nitrogen; N-NO_3_, concentration of nitrate nitrogen; TN, concentration of total nitrogen; P-PO_4_, concentration of phosphate phosphorus; TP, concentration of total phosphorus.

In all lakes, changes in the biological parameters were reflected in the general trend of the mean values of the dissolved and total fractions of biogenic compounds. The mean concentrations of the dissolved fractions of nitrogen (N-NH_4_ and N-NO_3_) and phosphorus (P-PO_4_) decreased, whereas the total fraction (TN and TP) increased with the growth of *P. rubescens* in the metalimnion of the studied lakes (Figures 5, 6). The mean concentration of N-NH_4_ of *ca.* 0.25 mg L^−1^ was similar in all lakes. Conversely, the mean concentration of N-NO_3_ varied between lakes and was about 0.6 mg L^−1^, 0.8 mg L^−1^, and 1.4 mg L^−1^ in lakes Rogóźno, Piaseczno and Krasne, respectively (Figures 6A,C,E). The mean values of P-PO_4_ differed slightly between lakes and were approximately 0.007–0.012 mg L^−1^ (Figures 6B,D,F). The highest mean values of TN in the metalimnion were noted in lakes Rogóźno in 2006 and Piaseczno in 2014 (*ca.* 3.0 mg L^−1^), whereas the lowest in Lake Krasne in 2010 (*ca.* 2.5 mg L^−1^, Figures 6A,C,E). The mean values of TP sharply increased in the metalimnion and reached the values of *ca.* 0.08 mg L^−1^ in lakes Krasne and Piaseczno, while in Lake Rogóźno were almost two times higher and reached *ca.* 0.15 mg L^−1^ (Figures 6B,D,F).

A clear trend in the vertical distribution of *P. rubescens* biomass was observed in all lakes. In general, the upper water layers from 0 to 5 m in lakes Rogóźno and Krasne or from 0 to 8 m in Lake Piaseczno were almost devoid or only with a small share of the filaments of *P. rubescens*. Contrarily, the water layers in metalimnion were dominated by its biomass (Figures 3G–I). The highest values of biomass of *P. rubescens* were always recorded in mid-June (13^th^–24^th^ June of the individual years) at the beginning of stable stratification periods, i.e., *ca.* 45 mg L^−1^ at a depth of 6 m in Lake Rogóźno (the highest value from all measurements that coupled with the highest values of chlorophyll-*a*, *ca.* 91 μg L^−1^) or *ca.* 16 mg L^−1^ at a depth of 6 m in Lake Krasne and *ca.* 14 mg L^−1^ at a depth of 11 m in Lake Piaseczno (Figures 3G–I). In light overexposure, the population of *P. rubescens* in all the studied lakes changed its position to actively migrate down or up the metalimnion layer. Hence, the highest biomass values were often found at different depths, even at biweekly intervals (Figures 3G–I).

### 3.4 The end of *Planktothrix rubescens* bloom

The development of *P. rubescens* in all studied lakes just suddenly ended in September when the thermal stratification started to be weaker and the mixing conditions reached the bottom of metalimnion – 8 m in lakes Rogóźno and Krasne and 12 m in Lake Piaseczno. The total biomass of phytoplankton significantly decreased and varied from 1.2 to 6.1 mg L^−1^, whereas the niche previously occupied by *P. rubescens* was dominated by the new species. In Lake Rogóźno, as in the winter season, by the cryptomonads *C. marssonii* and *C. curvata,* and in the other two lakes by filamentous cyanobacteria – in Lake Krasne by *Planktolyngbya limnetica*, which was already present in the metalimnion of this lake in August, and in Lake Piaseczno by *Limnothrix planctonica* (Woloszynska) Meffert and *Aphanizomenon gracile* Lemmermann.

### 3.5 *Planktothrix rubescens* biomass concerning biological and physicochemical parameters during its annual occurrences

**Figure 7:**
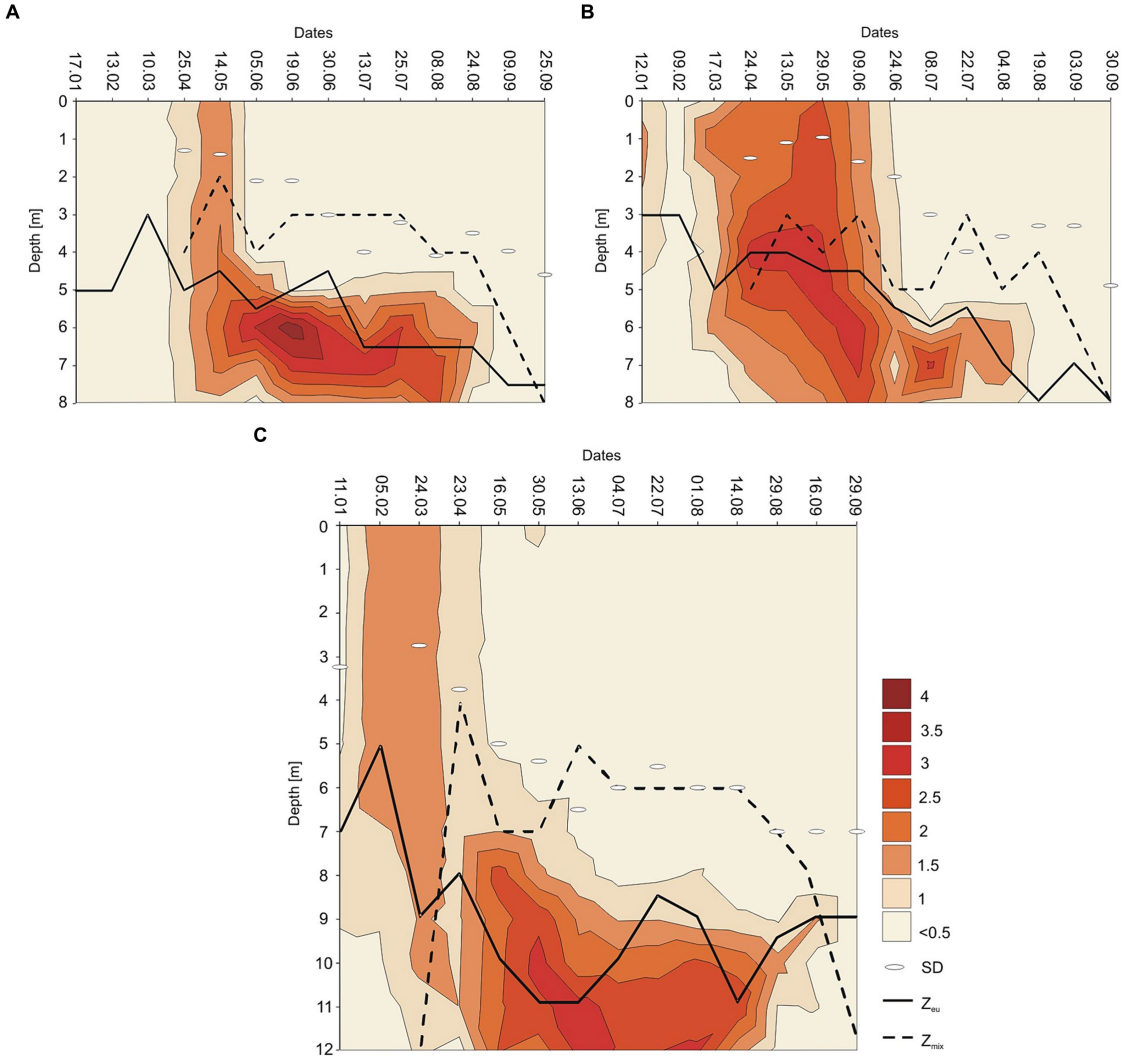
Contour plot of the annual distribution of the *Planktothrix rubescens* biomass as ln (x + 1) with euphotic depth (Z_eu_ in m, continuous line), mixing depth (Z_mix_ in m, dashed line), and Sechi Disk visibility (SD) in Lake Rogóźno in 2006 **(A)**; in Lake Krasne in 2010 **(B)**; in Lake Piaseczno in 2014 **(C)**.

The annual appearance of *P. rubescens* in the studied lakes was directly related to thermal and light conditions changes. In general, the first appearance of *P. rubescens* with different values of biomass were recorded in winter (January–March) just below the ice cover and the highest values of its biomass were noted in summer (June–August) in the metalimnion layer (Figure 7). During spring periods (23rd April–9th June), water transparency measured with Secchi Disk (SD) in lakes Rogóźno and Krasne was only 1–2 m, which corresponded to the smaller range of the euphotic zone (4–5.5 m). Such conditions affected the migration of *P. rubescens* into the upper 4–5 m water layers, which were susceptible to mixing (Figures 7A,B). In Lake Piaseczno, the SD values in spring were much higher (4–5.5 m), which influenced the greater range of the euphotic zone (8–11 m) and thus the presence of *P. rubescens* in deeper water layers outside the mixing zone (Figure 7C). The ecological niche in the metalimnion, once occupied in spring, remained a habitat for the development of *P. rubescens* throughout the summer stratification period, in which the highest values of biomass of this cyanobacterium were found at or below the euphotic zone at very low light intensities, PAR 0.6–27 μmol m^−2^ s^−1^ that constituted 0.05–1% of surface light intensities. This situation persisted until the end of August (Figures 3G–I). During the beginning of September, the metalimnetic bloom of *P. rubescens* underwent a complete breakdown, which corresponded to a disruption in the stability of thermal stratification caused by an increase in the range of the mixing zone in all lakes (Figure 7). Nevertheless, its annual development, lasting 8 months, had a significant impact on phytoplankton composition; when the biomass of *P. rubescens* reached *ca.* 2.5, 3.0, and 1.0 mg L^−1^ in Lakes Rogóźno, Krasne, and Piaseczno, respectively, its dominance in the total biomass of phytoplankton was at a level higher than 60% (Supplementary Figure S2). The high proportion of *P. rubescens* in the total biomass of phytoplankton affected the decrease in species richness and biodiversity (Shannon-Wiener index, H′), as well as evenness (Pielou’s index), which were within a similar range in all lakes. The values of H′ were higher in the epilimnion (1.5–2.7) than those in the metalimnion (0.7–1.5). Similarly, the evenness index and species richness ranged from 0.5–0.7 and 19–40 and 0.2–0.6 and 8–30 in the epilimnion and metalimnion, respectively. Hence, in all the studied lakes, negative Spearman correlations at a high level of significance (*p* < 0.001) were observed between *P. rubescens* biomass and these parameters (Table 2). Therefore, the biomass of *P. rubescens* was strongly positively correlated with biological factors (total biomass of phytoplankton and concentration of chlorophyll-*a*) and negatively correlated (*p* < 0.001) with the measured values of physical factors, such as light intensity (PAR) and water temperature (Table 2). The biomass of *P. rubescens* was also correlated with the chemical parameters. In general, low negative correlations, at a significance level of *p* < 0.05, were found with the dissolved fractions of nitrogen (N-NH_4_ and N-NO_3_) and phosphorus (P-PO_4_), whereas the values of the total fractions (TN and TP) correlated positively (p < 0.001) with biomass. Only in Lake Krasne was the lack of correlation with TN and P-PO_4_ noted (Table 2).

**Table 2:** Spearman rank correlation matrix (*r*_s_) of the biomass of *Planktothrix rubescens* and selected environmental parameters (bold numerals for *p* < 0.001; bold italic numerals for *p* < 0.05).

**Parameters**	**Lake** **Rogóźno (*n* = 126)**	**Lake** **Krasne** **(*n* = 126)**	**Lake** **Piaseczno (*n* = 182)**
Depth	**0.36**	0.10	**0.47**
Total nitrogen (TN)	**0.57**	−0.07	**0.29**
Ammonium nitrogen (N-NH_4_)	***−0.28***	***−0.25***	**−0.31**
Nitrate nitrogen (N-NO_3_)	***−0.26***	***−0.21***	**−0.43**
Phosphate phosphorus (P-PO_4_)	**−0.34**	0.08	**−0.32**
Total phosphorus (TP)	**0.36**	**0.71**	**0.72**
Photosynthetic Active Radiation (PAR)	**−0.31**	**−0.28**	**−0.54**
Temperature of water	**−0.36**	**−0.27**	**−0.70**
Concentration of chlorophyll-*a*	**0.86**	**0.70**	**0.76**
Total biomass of phytoplankton	**0.87**	**0.62**	**0.72**
Shannon-Wiener diverity index	**−0.58**	**−0.36**	**−0.72**
Pielou evenness index	**−0.56**	**−0.42**	**−0.64**
Number of species	**−0.37**	***−0.21***	**−0.62**

## 4 Discussion

Most climate warming scenarios indicate an increasing temperature trend, which is particularly significant during winter; this is directly related to the presence or absence of snow/ice cover and the consequent disruption of thermal water conditions during winter periods (Samuelsson, 2010; Dokulil, 2016; Woolway et al., 2020). The deep lakes of temperate zones, where the increase in surface water temperature during winter is the highest, are particularly susceptible (Dokulil et al., 2010). This trend was also observed in the lakes studied in eastern Poland under the influence of a temperate climate (Figure 1). Water warming directly impacts the functioning of aquatic organisms, especially small primary producers. One such species is *P. rubescens*, whose blooms are frequently recorded in various European lakes (Jacquet et al., 2005; Ernst et al., 2009; Dokulil and Teubner, 2012; Posch et al., 2012; Copetti et al., 2017). However, its response to changes and development during winter can vary depending on the location and characteristics of the lake. Thus, the same trend in climate change can generate differences in the appearance and pattern of *P. rubescens* development, especially during winter, which are important for establishing a novel population; this is particularly evident in alpine lakes where blooms of this cyanobacterium have been recorded permanently for many decades, with some data dating back to the 19th century (Walsby and Schanz, 2002; Jacquet et al., 2005; Legnani et al., 2005; Ernst et al., 2009; D’Alelio et al., 2011; Dokulil and Teubner, 2012; Hingsamer et al., 2014; Yankova et al., 2016; Copetti et al., 2017; Fernández Castro et al., 2021; Knapp et al., 2021; Carratalà et al., 2023). Recent studies of alpine lakes have indicated that warming water masses have multidirectional effects on *P. rubescens* bloom dynamics.

Moreover, a paradox related to the positive impact of climate warming has been found: a small population of *P. rubescens* after periods of warmer winters produces a more intense bloom during periods of thermal stratification than a large population after periods of cold winters (Knapp et al., 2021). Temperate lakes in the lowlands appear to respond differently to such changes, as evidenced by reports of occasional blooms of *P. rubescens*, often recorded at different time intervals (Krupa and Czernaś, 2003; Padisák et al., 2003; Salmaso and Padisák, 2007; Kröger et al., 2023). Some reports suggest that *P. rubescens* blooms in winter may have been influenced by prolonged periods of ice in the year before the bloom occurred (Padisák et al., 2003). Our study extends this conjecture to a certain extent. During the 24 years considered (2000–2023), *P. rubescens* was found only three times, and its appearance was associated with extremely low temperatures in January. In eastern Poland, such conditions are associated with Arctic or polar continental air masses heralding prolonged periods of ice cover, ensuring the thermal stability of lake waters during winter. The blooms discussed in this study, separated by 4-year intervals, occurred under such conditions and began with low intensity during winter periods; the lowest values of *P. rubescens* biomass (below 0.6 mg L^−1^) were found in Lake Rogóźno in 2006, while much higher values (often above 2 mg L^−1^, Figures 3A–C) were found in Lakes Krasne and Piaseczno. In deep alpine lakes, climate warming has a limiting effect on the mixing depth, significantly reducing the internal release of biogenic compounds from the hypolimnetic layers and decreasing their availability in the upper water layers. This process limits the development of eukaryotic phytoplankton, such as diatoms and cryptomonads, and increases the development of prokaryotic species, such as *P. rubescens* (Yankova et al., 2017; Knapp et al., 2021). This effect was not observed in the temperate lakes studied, in which an even distribution of nutrients was observed throughout the water column in winter. This is probably influenced by the almost 10-fold lower maximum depth of the studied lakes (25–39 m) compared to that of the Alps, which allows the release of internal nutrients deposited in the bottom sediments during the autumn mixing of waters preceding the occurrence of ice periods. Thus, our observations suggest that winter appearances of this cyanobacterium in temperate lakes, unlike those in alpine lakes, are related to extremely cold winter coupled with long-lasting periods of ice cover. Owing to their occasional character and relatively shallow depth, blooms are not limited by the availability of nutrients.

This study also showed that physical factors affected by ice phenology, such as thermal and light conditions, are important for the development of the winter population of *P. rubescens*. Under the thick snow/ice cover, specific, albeit stable conditions of dim light climate occurred, which combined with reverse thermal stratification to support the vertical migration of *P. rubescens*, with highest values of biomass were often found just under the ice cover in lakes Rogóźno and Krasne in 2006 and 2010, respectively. The bloom recorded in Lake Piaseczno in the winter of 2014 occurred during a warmer period, and the biomass of *P. rubescens* was often evenly distributed in the 0–6 m water layer. Nevertheless, polar continental air masses were also recorded during this winter, resulting in an ice cover that lasted almost 2 months. In addition, even if no ice cover was present in winter, the light conditions were moderate because of the long-lasting cloudy and rainy weather, which effectively hindered solar radiation and indirectly protected the biomass of *P. rubescens* against photoinhibition. Since 2014, all three lakes have been monitored yearly during winter and summer. Still, the presence of *P. rubescens*, which we associate with unusually warm winters, has not been recorded once. The paradox presented in alpine lakes by Knapp et al. (2021) related to the more intensive development of *P. rubescens* in the metalimnion in summer after a period of weaker development of its population during warmer winters in a sense also occurred in the studied temperate lakes. However, this is not regarding the type of winter (warm or cold) but of the appearance of a much more intense bloom of *P. rubescens* during summer stratification after a winter inoculum with very low biomass, which occurred in Lake Rogóźno in 2006 and did not occur in the other lakes (Figures 3A–C,G). Low biomass persisted in this lake during the spring mixing period (Figure 3D); however, in late spring (9^th^ June), its population visibly increased in the metalimnion after the establishment of thermal stratification of the waters. The highest value of *P. rubescens* biomass (44.8 mg L^−1^) was recorded 2 weeks later at a depth of 6 m. Similar dynamics in population development, although with lower biomass values, have also been observed in other temperate lakes (Krasne and Piaseczno). This bloom scheme reflects the three phases of the annual development of *P. rubescens* in the alpine Lake Mondesee (Dokulil and Teubner, 2012). From vertical migration to the metalimnion, *P. rubescens* remained until the end of the thermal stratification period (end of August), maintaining its biomass at approximately 15 mg L^−1^. Thus, we conclude that the presence of *P. rubescens* in temperate lakes during the winter period, regardless of its biomass, is a guarantee of its bloom during the summer period, often with high intensity.

Therefore, monitoring of deep temperate lakes where *P. rubescens* has previously appeared should take place during winter periods, as this provides an opportunity to plan future research during the summer stratification period. Based on the available literature, only one lake, Piaseczno, had a previous occurrence of *P. rubescens*, which was more than 20 years earlier (1986–1996); its appearance was of low intensity and it was occasionally found (every few years). In addition, the studies were carried out only during the growing season (spring-autumn) and at large time intervals (one sample representative of the season), thus unintentionally ignoring the important role of ice phenology in creating the inoculum for further annual development of *P. rubescens* populations (Krupa and Czernaś, 2003). Only once, in 1989, a bloom of this cyanobacterium was reported in Lake Piaseczno and was linked with the increase in nutrients level; however, the study focused mostly on its effect on the qualitative and quantitative structure of phytoplankton. Therefore, it was concluded that the presence of this species was a symptom of the eutrophication of Lake Piaseczno (Wojciechowska and Krupa, 1992; Krupa and Czernaś, 2003). However, after the blooms disappeared, the lake returned to a mesotrophic state.

The summer period in the studied lakes, along with stable thermal stratification, presented a typical course of *P. rubescens* bloom dynamics in various European lakes (Micheletti et al., 1998; Walsby and Schanz, 2002; Jacquet et al., 2005; Legnani et al., 2005; Dokulil and Teubner, 2012; Posch et al., 2012; Yankova et al., 2016; Fernández Castro et al., 2021; Knapp et al., 2021; Moiron et al., 2021; Carratalà et al., 2023). Notably, different depths at which this species developed were recorded in the studied lakes during summer. The major reason was the location and thickness of the metalimnion layer, which was 4–8 m in lakes Rogóźno and Krasne and 7–12 m in Lake Piaseczno. The occurrence of the bloom of *P. rubescens* on higher depths in Lake Piaseczno was also influenced by the high transparency of the waters (5.5–6.5 m) and the large range of the euphotic zone (8.5–11 m), which were much deeper compared to the other lakes. Nevertheless, a similar pattern of vertical migration of this species into the metalimnion layer and the movement of its populations within this layer in response to changing light conditions has been recorded in all lakes during summer, which is called light-driven buoyancy regulation (Micheletti et al., 1998; Dokulil and Teubner, 2012; Knapp et al., 2021). This is mainly due to the need to avoid mixing of the water and too high light intensity, which was particularly noticeable at the point of maximum bloom recorded in lakes Rogóźno and Krasne at depths of 6–7 m and in the much more transparent Lake Piaseczno almost twice as deep at a depth of 11 m (Figures 3G–I, 7). The highest biomass of *P. rubescens* in the metalimnion of studied lakes occurred at very low values of photosynthetically active radiation (PAR, 0.6–27.1 μmol m^−2^ s^−1^) and in low values of water temperature (8–12°C), which was confirmed by strong negative correlation with these parameters (Table 2). Such conditions were often measured below the euphotic zone in the dim light layer, close to 0.1% of light intensity, and are similar to the values noted during summer periods in alpine lakes by other authors (Micheletti et al., 1998; Walsby and Schanz, 2002; Jacquet et al., 2005; Dokulil and Teubner, 2012; Fernández Castro et al., 2021; Knapp et al., 2021; Carratalà et al., 2023); this suggests the existence of a certain summer bloom pattern independent of the type of lake and climatic zone in which it is located.

The values of dim light climate in the studied lakes, in which *P. rubescens* developed well in the summer periods, were comparable to those noted under thick snow/ice cover during severe winters. The role of dim light was also confirmed in a laboratory experiment that demonstrated the positive effect of low quantities of green light and the strong negative effects of high intensities of white light on the development of *P. rubescens* (Oberhaus et al., 2007). However, the dim light climate in winter is used to establish an inoculum for the population so that later in summer, similar light conditions represent the optimum conditions for an intensive metalimnetic bloom. In addition the bloom of *P. rubescens* was associated with almost complete dominance in the phytoplankton, often reaching 60–90%. Similar dominance values were also found in Alpine Lake Pusiano (Legnani et al., 2005). The high proportion of *P. rubescens* in the phytoplankton of the studied lakes was most likely due to the allelopathic effect of this species, which had a limiting effect on the total biomass of phytoplankton, not only in the metalimnion but also in the upper water layers (Kurmayer et al., 2016). This activity ensured an optimal light climate for blooms in the metalimnion. The allelopathic interactions of this cyanobacterium are related to the secretion of toxins (microcystins) and secondary metabolites, described previously in mixed culture experiments (Oberhaus et al., 2008). In this study, the allelopathic effect of *P. rubescens* populations was also associated with a significant decrease in the biodiversity and species richness of the phytoplankton community, as confirmed by Spearman correlations (Table 2). Similar decreases in phytoplankton biodiversity indices were found earlier in the bloom layer of Lake Pusiano (Legnani et al., 2005). Recent reports from Alpine Lake Geneva also suggest a limiting effect of the biomass of *P. rubescens* on the taxonomic and functional diversity of bacterioplankton (Carratalà et al., 2023).

Chemical factors, especially the dissolved nitrogen and phosphorus fractions, influenced the development of *P. rubescens* in the temperate lakes studied, as confirmed by Spearman’s correlations (Table 2). Particularly in summer, decreases in dissolved nutrients (N-NO_3_, N-NH_4_ and P-PO_4_) were found in the bloom layer in lakes Rogóźno and Piaseczno, which was not so evident in lake Krasne (Figure 6). This may be due to the higher proportion of other cyanobacterial species, *Limnothrix planctonica* and *Planktolyngbya limnetica* in the metalimnion layer of this lake. However, during annual blooms, the pool of available nutrients did not limit the development of the *P. rubescens* population. We attribute this to the high concentrations of dissolved nutrients recorded in winter and in the upper water layers of the studied lakes during summer. Similarly, high concentrations of P-PO_4_ have been recorded in alpine lakes during the winter (Legnani et al., 2005). There are also mechanisms by which *P. rubescens* can utilize, in addition to the nitrate fraction, the ammonium fraction of nitrogen, which increases the pool of available nitrogen compounds and gives it a competitive advantage over other algae (Köster and Jüttner, 1999). Furthermore, the low concentrations of phosphate (P-PO_4_) available in the water of the studied lakes (4–18 ug L^−1^) probably did not limit the development of *P. rubescens*. Similar values of P-PO_4_ (0–10 ug L^−1^) were often found during its blooms in Dutch lakes, as well as in alpine and temperate lakes (Schreurs, 1992; Chorus et al., 2011; Moiron et al., 2021). Even in the limitation of P-PO_4_, *P. rubescens* can utilize organic forms of phosphorus due to the presence of alkaline phosphatase or other sources of phosphorus decomposed from organic matter by heterotrophic bacteria (Feuillade et al., 1990; Jacquet et al., 2005).

The development of *P. rubescens* promptly ended in early September, when the extent of the mixing zone increased rapidly, reaching the lower layer of metalimnion (8 m in lakes Rogóźno and Krasne, 12 m in Lake Piaseczno); this led to a breakdown of the population and the complete disappearance of this species from the studied water layers. The ecological niche left by *P. rubescens* was filled by species belonging to cryptomonads in Lake Rogóźno or other filamentous cyanobacteria in Lakes Krasne and Piaseczno. A similar scheme of complete breakdown of the population of *P. rubescens* and the development of other phytoplankton groups, diatoms, and chlorophytes in autumn has been found in temperate Lake Stechlin (Padisák et al., 2003; Salmaso and Padisák, 2007); this confirms the different dynamics of *P. rubescens* bloom in temperate lakes, which ends with the complete disappearance of its population with an undetermined moment of the next appearance. In alpine lakes, there are temporary decreases in the biomass of this species in autumn and the initiation of a new population in the following winter. Global warming has a positive influence on the development of blooms in alpine lakes, increasing water temperature, which reduces the range of the mixing zone and protects the gas vesicles in the filaments of *P. rubescens* from damage caused by high hydrostatic pressure during convective mixing, which in turn has a positive influence on the dispersion and buoyancy of this species and the initiation of a new population during the forthcoming winter (D’Alelio et al., 2011; Carey et al., 2012; Posch et al., 2012; Yankova et al., 2016, 2017; Fernández Castro et al., 2021; Knapp et al., 2021). Our research shows that such a phenomenon does not occur in temperate lakes in the lowlands. In contrast, although global warming causes similar increases in water temperatures in winter, it simultaneously reduces the occurrence of severe winters and the duration of ice cover, the presence of which is extremely important because of the establishment and persistence of *P. rubescens* populations during winter periods under stable ice cover. The lack of ice periods in winter often results in the mixing of water masses, which can persist continuously from autumn to spring, as it was at the turn of 2006 and 2007 in Lake Rogóźno when the mixing period lasted for 7 months. Such conditions negatively affect the possibility of initiating a new bloom of this cyanobacterium; however, they also support the development of other phytoplankton groups, such as diatoms (Lenard and Ejankowski, 2017).

Our study showed that the development of *P. rubescens* populations during the annual cycle in the temperate lakes studied was more influenced by physical conditions (ice phenology, low light intensity, and water temperature) than by chemical parameters (dissolved and total fractions of nitrogen and phosphorus). However, the following question arises: why did blooms not appear simultaneously in the studied lakes? We can only partially answer this question. One of the reasons for the absence of *P. rubescens* blooms in Rogóźno Lake in 2010 and 2014, when the blooms were found in the other lakes, was a significant change in the water color of this reservoir caused by a rapid rise in the water level (by more than 0.5 m), which took place in 2007; this led to the flooding of the peatlands adjacent to the lake and the release of deposited humic compounds, resulting in intense brownification of lake water. The effect of the color change was a drastic decrease in water transparency, a significant deterioration in light conditions, and consequently, a complete qualitative and quantitative remodeling of the phytoplankton community combined with the dominance of flagellate species (Lenard and Ejankowski, 2017). High watercolor is maintained in Lake Rogóźno to the present (almost 20 years), affecting the small range of the euphotic zone, which is even shallower than the mixing zone. Such conditions negatively affected the possibility of *P. rubescens* blooms in this lake, even during severe winter in 2010 and 2014, when blooms were noted in lakes Krasne and Piaseczno, respectively. The absence of this cyanobacterium in Lake Krasne in 2006 and 2014 was likely due to the occurrence of high biomass of diatoms, e.g., *Stephanodiscus medius*, and cryptomonads, e.g., *Cryptomonas curvata* and *Plagioselmis nannoplanctica* in winter periods (Lenard and Wojciechowska, 2013) and the frequent blooms of other filamentous cyanobacteria, e.g., *Limnothrix planctonica*, *Limnothrix redekei* and *Planktolyngbya limnetica*, in the upper water layers (0–4 m) during summer periods, which significantly reduced the light climate in the lake and the possibility of the development of *P. rubescens*. However, blooms did not occur in Lake Piaseczno in 2006 and 2010, when the phytoplankton biomass in winter was dominated by green algae such as *Botryococcus braunii*, *Closterium acutum* var. *variabile,* and cryptomonads, for example, *Cryptomonas* sp. (Wojciechowska and Lenard, 2014), whereas coccal cyanobacteria, such as *Aphanothece clatrata* and *Radiocystis geminata*, dominated the phytoplankton biomass in the upper water layers (0–8 m) during summer. Another question remains: What happens to *P. rubescens* filaments in temperate lakes during periods of prolonged absence of blooms? A similar dilemma regarding the return of the bloom after an absence of several years has appeared in Alpine Lake Bourget, but it has still not been clarified (Moiron et al., 2021). In our opinion, presumably during the autumn period, filaments are shortened, they lose the aerotopes, form hormogonia, and then relocate to the bottom sediments, which is characteristic of the population of *Planktothrix agardhii* (Poulíčková et al., 2004). It is likely that, in this form, the population waits until the establishment of a new winter inoculum only if favorable conditions occur in the lake from a certain climatic zone.

In conclusion, the blooms in temperate lakes in the lowlands present similar patterns of *P. rubescens* population development during the growing season (spring–summer) as in alpine lakes. However, these species differ significantly in the formation of winter inocula during annual blooms (Walsby et al., 1998; Salmaso, 2000; Moiron et al., 2021). An important role in this process is provided by the specific conditions of severe winters affecting lake ice phenology, which allow the initiation of the appearance of *P. rubesens* under ice cover. Our studies show that Global warming, manifested by the occurrence of increasingly warmer winters devoid of ice cover, has a negative impact on the possibility of establishing a new population of *P. rubescens* in temperate lakes during winter, which is not the case for alpine lakes (Yankova et al., 2017; Knapp et al., 2021; Moiron et al., 2021; Carratalà et al., 2023). The consequence of the lack of winter inoculum was the absence of *P. rubescens* throughout the year. In the lakes studied, blooms were found in 4 years intervals (2006, 2010, and 2014) that occurred over approximately 10 years, when severe winters with ice cover were noted as many as three times. Since the last bloom in Lake Piaseczno in 2014, another *ca.* 10 years have passed, during which no single occurrence of severe winters has been recorded, resulting in the absence of *P. rubescens*. Therefore, the occasional presence of this species in shallower temperate lakes seems particularly interesting, as it constitutes a counterpoint to the permanent blooms of this cyanobacterium found in very deep alpine lakes.

## Data availability statement

The raw data supporting the conclusions of this article will be made available by the authors, without undue reservation.

## Author contributions

TL: Conceptualization, Data curation, Formal analysis, Investigation, Methodology, Resources, Supervision, Validation, Visualization, Writing – original draft, Writing – review & editing. WE: Data curation, Investigation, Methodology, Writing – review & editing.

## Footnotes

^1^ www.tutiempo.net

## Supplementary material

Supplementary Figure S1 Relation, in the average values of minimum **(A)**, maximum **(B)**, and mean **(C)** temperatures of air in January 2000–2023, between the years with or without the presence of *Planktothrix rubescens*. 0, periods without *P. rubescens*; 1, periods with *P. rubescens*.

Supplementary Figure S2 The relative contribution of the biomass of *Planktothrix rubescens* to the biomass of total phytoplankton in Lake Rogóźno in 2006 **(A)**; in Lake Krasne in 2010 **(B)**; in Lake Piaseczno in 2014 **(C)**.
