# Supplementary figures and images for: The ice phenology as a predictor of Planktothrix rubescens bloom in vegetation season in temperate lakes
Source: Front Microbiol. 2024 Jun 26;15:1384435. doi: 10.3389/fmicb.2024.1384435 (PMC11233451; doi:10.3389/fmicb.2024.1384435)

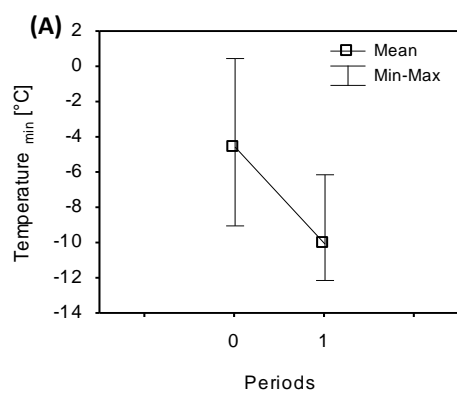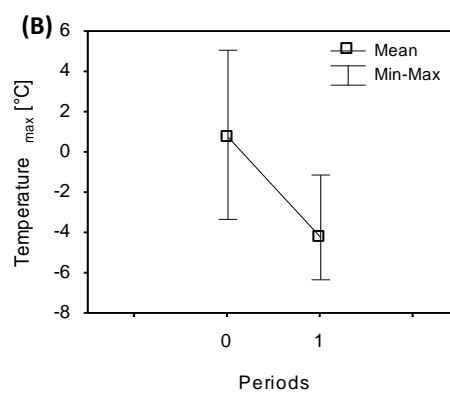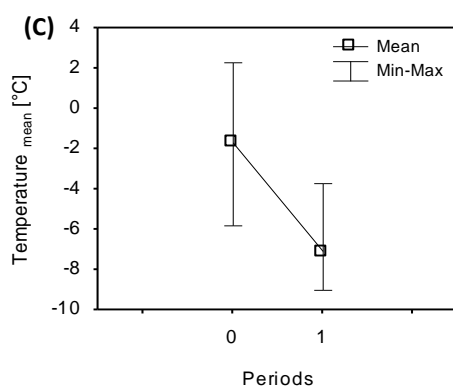

Supplement: Supplementary Figure S1 — Relation, in the average values of minimum (A), maximum (B), and mean (C) temperatures of air in January 2000–2023, between the years with or without the presence of Planktothrix rubescens. 0, periods without P. rubescens; 1, periods with P. rubescens. [file Data_Sheet_1.PDF]

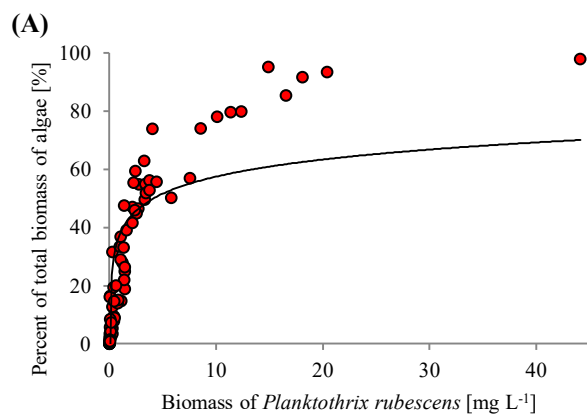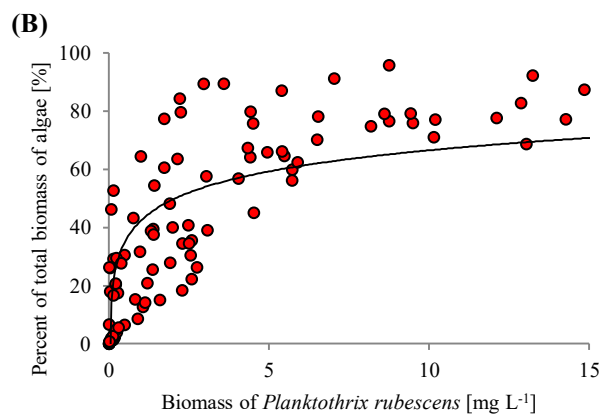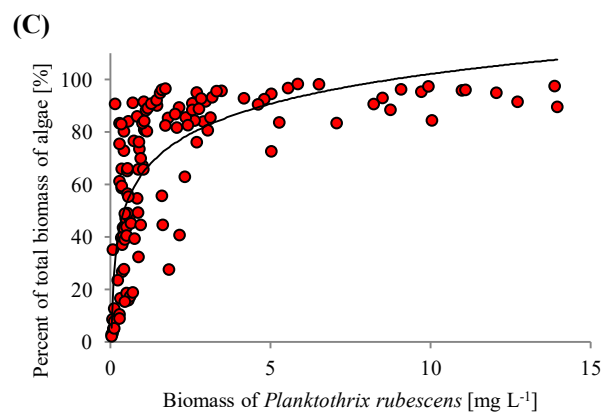

Supplement: Supplementary Figure S2 — The relative contribution of the biomass of Planktothrix rubescens to the biomass of total phytoplankton in Lake Rogóźno in 2006 (A); in Lake Krasne in 2010 (B); in Lake Piaseczno in 2014 (C). [file Data_Sheet_2.PDF]
